# Fractures of the Os Femoris

**Published:** 1829

**Authors:** 


33. *Fractures of the os Femoris.*—In the American Medical Recorder for January, 1829, we find a practical paper on fractures of the thigh bone, by an old and experienced physician of Massachusetts, Dr. Josiah Goodhue; in which he gives the following account of a method of making permanent extension of the limb, which he has long and successfully employed.

Let boards be sawn a suitable length to drop in upon the bed-cord, between the head and foot pieces of the bedstead, on which lay a good straw bed or mattrass and a blanket. Then take two common pillows, and after removing half of the feathers, lay them lengthwise upon the bed, in a position to receive the leg and thigh. Prepare the tail bandage, not quite as wide as the thigh is long, and place it properly upon the pillow on which the thigh is to rest. Place three pieces of narrow tape under each pillow; let a piece of planed board two inches and a half wide, be nailed to the outside of the footpiece of the beadstead, so that the top of it will be two inches higher than the toes of the patient, when the foot lies upon the heel. Let a large silk handkerchief be doubled about three inches wide, and wound closely around the ankle near the foot, and sown with strong thread so that it will not give way in any part. Now after the board upon which the patient rests is placed on the side of the bed, let the surgeon take hold of the broken limb, with other assistance sufficient to lay the patient upon his back, his thigh and leg resting on the pillows. Let two pieces of strong wide tape be sewed to the handkerchief, one on the outside and the other on the inside of the ankle, the tape ought to be a foot and a half long.

The surgeon will now be prepared to extend the limb and adjust the bone, which ought to be done with great moderation and tenderness, as I know from long experience, that violence is quite unnecessary. Then let a strong man stand at the head of the bed, and place his hands with firmness under the patient's arms, so that the body may not be drawn down when the extension is made. I should advise the surgeon to make the first extension in a very slow, firm, and gradual manner, he will find that by continuing the extension for two or three minutes, he will gain more, than by ten times the force applied with sudden violence. I would now give the limb into the hands of a trusty assistant, and place my hands upon the fracture. As it is impossible to give directions with regard to the coaptation of the ends of the bone, I shall leave that part to the ingenuity of the operator, he bearing in mind that hard pinching is never necessary. The tapes attached to the handkerchief may now be tied with firmness round the footpiece. A double cloth may be wet with a solution of the sugar of lead or rum and water, and applied to the thigh, the tail bandage being nicely, but not tightly folded. Then let the foot of the bed be raised, so as to place two bricks flatwise, under each post. The extension may now be discontinued. It will be seen that the patient lies upon a gently inclined plane, with his foot firmly fixed, which is all that is necessary (with constant care) to keep the bone in place. Splints of every kind, in this way of proceeding, are entirely useless.

